# H_2_O_2_-responsive and plaque-penetrating nanoplatform for mTOR gene silencing with robust anti-atherosclerosis efficacy†
†Electronic supplementary information (ESI) available: Detailed experimental procedures, supporting tables and figures. See DOI: 10.1039/c7sc03582a


**DOI:** 10.1039/c7sc03582a

**Published:** 2017-10-27

**Authors:** Wen Gao, Yujie Zhao, Xiang Li, Yuhui Sun, Michelle Cai, Wenhua Cao, Zhenhua Liu, Lili Tong, Guanwei Cui, Bo Tang

**Affiliations:** a College of Chemistry, Chemical Engineering and Materials Science , Collaborative Innovation Center of Functionalized Probes for Chemical Imaging in Universities of Shandong , Key Laboratory of Molecular and Nano Probes , Ministry of Education , Institute of Biomedical Sciences , Shandong Normal University , Jinan 250014 , P. R. China . Email: tangb@sdnu.edu.cn; b Faculty of Science , Western University , London , Ontario N6A5B7 , Canada

## Abstract

The mammalian target of rapamycin (mTOR) that controls autophagy and lipid metabolism is pivotal for atherosclerosis initiation and progression. Although blocking the mTOR function with rapamycin and its analogs may stimulate autophagy and consequently attenuate lipid storage and atherosclerotic lesions, only limited success has been achieved in clinical applications due to the unsatisfactory efficacy and safety profiles. In this study, we engineered a cerium oxide nanowire (CeO_2_ NW)-based RNA interference (RNAi) oligonucleotide delivery nanoplatform for the effective silencing of mTOR and treatment of atherosclerosis. This nanoplatform is composed of the following three key components: (i) a stabilin-2-specific peptide ligand (S2P) to improve plaque targeting and penetration; (ii) polyethylene glycosylation (PEGylation) to extend *in vivo* circulation time; and (iii) a high aspect ratio CeO_2_ core to facilitate endosome escape and ensure “on-demand” release of the RNAi payloads through competitive coordination of cytosolic hydrogen peroxide (H_2_O_2_). Systemic administration of the nanoplatforms efficiently targeted stabilin-2-expressing plaque and suppressed mTOR expression, which significantly rescued the impaired autophagy and inhibited the atherosclerotic lesion progression in apolipoprotein E-deficient (ApoE^–^/^–^) mice fed with a high-fat diet. These results demonstrated that this H_2_O_2_-responsive and plaque-penetrating nanoplatform can be a potent and safe tool for gene therapy of atherosclerosis.

## Abstract 2

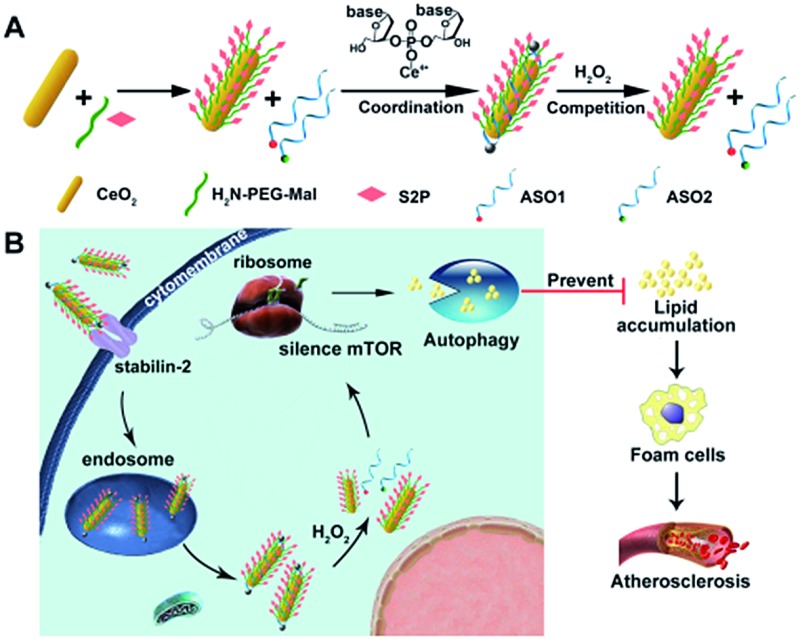
A H_2_O_2_-responsive and plaque-penetrating S2P–CeO_2_–ASOs nanoplatform was developed for the effective silencing of mTOR and treatment of atherosclerosis.

## Introduction

Atherosclerosis is the leading cause of cardiovascular disease and stroke which increasingly threatens human health worldwide.^1^,^2^ The initiating event in atherosclerosis is the accumulation of lipids within the arterial wall that leads to the formation of vascular smooth muscle cell (VSMC)/macrophage (Mφ)-derived foam cells.^3^–^5^ Recently, the mammalian target of rapamycin (mTOR) has drawn more attention to atherosclerosis as it has an important role in the regulation of autophagy.^6^,^7^ Autophagy is a protective and life-sustaining process by which lipid droplets within the VSMC/Mφ are sequestered in double-membrane vesicles and degraded *via* lysosomal acid lipase after fusion with lysosomal compartments.^8^–^10^ Inhibiting mTOR function with rapamycin or its derivative everolimus activates the autophagy process and impedes foam cell formation, suggesting a promising target for the treatment of atherosclerosis.^11^–^13^ However, only limited success has been achieved in clinical applications of mTOR inhibitors due to their poor solubility and low bioavailability.^14^,^15^ Moreover, rapamycin does not perturb all mTOR functions; at least two distinct multiprotein complexes of mTOR exist within cells, only one of which is sensitive to rapamycin.^16^,^17^ Therefore, the exploration of more efficient strategies for mTOR inhibition is highly desirable.

RNA interference (RNAi) using an antisense oligonucleotide (ASO) or short-interfering RNA (siRNA) is an attractive alternative to inhibiting otherwise intractable therapeutic targets.^18^,^19^ Effective knockdown occurs once an ASO or siRNA strand is incorporated into the RNA-induced silencing complex, which is then guided to the target mRNA sequence to be excised.^20^,^21^ This ensures that the “nondruggable” target proteins are no longer expressed within the cell.^22^ Although this strategy has been effective *in vitro*, delivery of therapeutic ASOs or siRNAs into diseased tissues or cells of patients remains a challenge. For atherosclerosis therapy, the barriers to effective *in vivo* siRNA delivery mainly include serum nuclease degradation, atherosclerotic plaque targeting, plaque and cell membrane penetration, escape from the endosome, and release of ASOs or siRNAs in the cytoplasm.^23^ Recent advances in nanotechnology make it possible to overcome the extracellular barrier by packaging ASOs or siRNAs into nanoparticles. Based on their large surface areas, intrinsic properties and nanoscale effects, nanoparticles can accommodate a variety of ASOs or siRNAs with a high loading efficiency^24^,^25^ and act as a protective barrier against the enzymatic environment outside of the cells, prolonging their half-life in the circulation.^26^–^29^ In addition, the defective and immature nature of the blood vessels surrounding atherosclerotic plaques allows ASO- or siRNA-loaded nanoparticles to extravasate the leaky blood vessels and accumulate in the plaques, relying on the enhanced permeation and retention (EPR) effect.^30^ However, the efficient delivery and release of the ASOs or siRNAs to the target VSMC/Mφ against non-specific cellular uptake, subsequent endosomal entrapment and lysosomal degradation remain an intractable problem. In order to successfully inhibit the mTOR function with ASOs or siRNAs *in vivo*, the nanoparticle delivery platform should be tailored to exhibit the following features: (1) good biocompatibility, (2) a long blood circulation time and high EPR effect, (3) plaque-penetrating ability and VSMC/Mφ-targeting specificity, (4) an efficient endosomal escape ability and the “on-demand” release of active ASOs or siRNAs into the cytoplasm, and (5) an imaging function for monitoring the release profile and therapeutic efficacy.

**Scheme 1:**
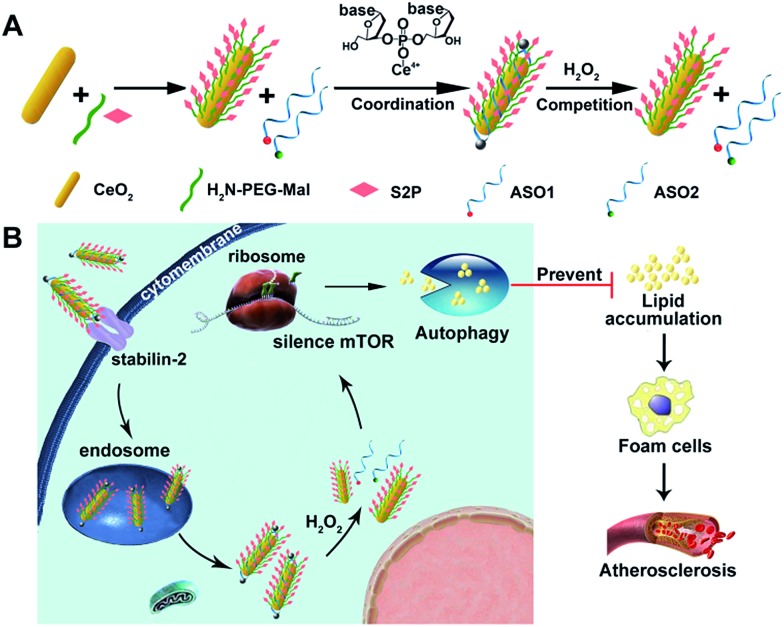
(A) Molecular structures of the H_2_O_2_-responsive and plaque-penetrating nanoplatform, S2P–CeO_2_–ASOs. (B) Illustration of the S2P–CeO_2_–ASOs nanoplatform for targeted mTOR gene silencing to attenuate atherosclerosis.

Herein, we developed an engineered cerium oxide nanowire (CeO_2_ NW)-based RNAi oligonucleotide delivery nanoplatform capable of satisfying all of the above-mentioned criteria and demonstrated its high efficiency in suppressing mTOR expression and atherosclerosis progression. This nanoplatform consisted of a high aspect ratio CeO_2_ NW functionalized with PEG (MW 2000, surface protection moiety^31^) and a plaque-penetrating peptide (S2P, stabilin-2-specific peptide ligand^32^). Two different mTOR ASOs naturally wrapped around the S2P-PEGylated CeO_2_ NW through metal coordination^33^ between phosphate groups in DNA and Ce ions at the particle surfaces (Scheme 1A). The resulting S2P–CeO_2_–ASOs nanoplatform exhibited an excellent biocompatibility and prolonged blood circulation lifetime. It also had the ability to specifically target and penetrate stabilin-2-overexpressing VSMCs, both in the culture and in the atherosclerotic plaques of apolipoprotein E-deficient (ApoE^–^/^–^) mice. Once inside the cell, the high aspect ratio CeO_2_ core was able to pierce the endosomal membrane, thus facilitating the loaded ASOs escape from the endosome prior to lysosomal degradation. As demonstrated in previous atherosclerosis studies,^34^,^35^ plaque cells exhibit a higher level of cytosolic reactive oxygen species (ROS), particularly hydrogen peroxide (H_2_O_2_). In the presence of H_2_O_2_, the absorbed ASOs are liberated from the CeO_2_ surface due to the stronger binding ability between peroxo and Ce^4+^ compared to that between phosphate oxygen and Ce^4+^.^36^,^37^ Fluorescently tagged ASOs were used to monitor the endosome rupture and cytosolic H_2_O_2_-triggered release. Targeted delivery and “on-demand” release of ASOs to high-fat diet-fed ApoE^–^/^–^ mice efficiently depleted mTOR expression and rescued the impaired autophagy, which led to a dramatic reduction in foam cell formation and atherosclerotic lesion progression (Scheme 1B). These results suggested that the S2P–CeO_2_–ASOs nanoplatform can be a potential new therapeutic option for atherosclerosis.

## Results and discussion

### Preparation and characterization of the S2P–CeO_2_–ASOs nanoplatform

**Fig. 1:**
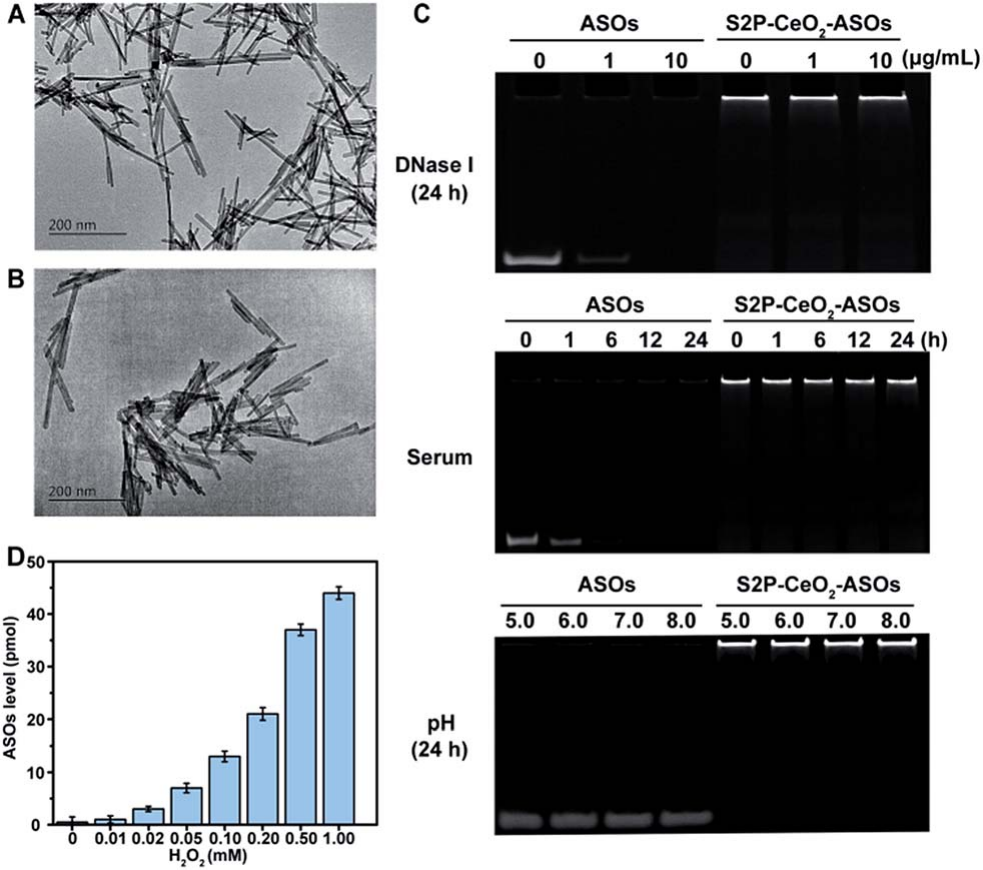
Characterizations of the S2P–CeO_2_–ASOs nanoplatform. TEM images of (A) CeO_2_ NWs and (B) S2P–CeO_2_–ASOs incubated in PBS buffer (pH 7.4). Scale bar = 200 nm. (C) Stability of free and S2P–CeO_2_–ASOs-encapsulated ASOs in DNase I, FBS-supplemented DMEM, and HEPES buffer (pH range = 5.0 to 8.0) for the indicated time at 37 °C. (D) H_2_O_2_-triggered release of ASOs from S2P–CeO_2_–ASOs.

To realize our design, CeO_2_ NWs were synthesized *via* a modified hydrothermal method using Ce(NO_3_)_3_ as the Ce source.^38^ A high aspect ratio CeO_2_ NW with a dimension of 100–150 nm was selected as the core unit, which is the optimal size for EPR-mediated targeting and endosomal escape and allows for maximal RNAi oligonucleotide loading. Transmission electron microscopy (TEM) showed that the obtained CeO_2_ NWs presented a smooth surface with the length being 130 nm and width being 9 nm (Fig. 1A). Their X-ray diffraction (XRD) pattern in Fig. S1† indicated that these particles were composed of CeO_2_ of the typical fluorite cubic structure (JCPDS card 34-0394, space group *Fm3m*).^39^ The zeta potential of CeO_2_ NWs in PBS (pH 7.4) was determined to be –14.2 ± 0.4 mV, which allowed for electrostatic interactions with positively charged maleimide–PEG–amine (MW 2000). The reversed zeta potential of 2.2 ± 0.5 mV indicated that PEG was successfully coated onto the CeO_2_ core surface. The plaque-penetrating peptide S2P, Cys-Arg-Leu-Thr-Leu-Thr-Val-Arg-Lys-Cys, was further grafted to the PEG-capped CeO_2_ by thioether bonds between the thiol groups on Cys and maleimide groups on PEG, resulting in a much higher positive zeta potential (4.3 ± 0.3 mV). The S2P-PEGylated CeO_2_ NW was then wrapped with mTOR-specific ASO1 and ASO2 (sequences are described in Table S1†) *via* metal coordination between phosphate groups (PO_4_^–^) in DNA and Ce ions (Ce^4+^) at the particle surfaces. The loading efficiency of the ASOs was calculated using a NanoDrop spectrophotometer. The average number of ASOs loaded in S2P-PEGylated CeO_2_ NWs was estimated to be 435 DNA/NW (Table S2†). We also evaluated the distribution of ASO1 and ASO2 in the coloaded particles by quantitative real-time PCR (qRT-PCR) analysis. In three different batches, the results indicated that coloading of ASO1 and ASO2 in S2P-PEGylated CeO_2_ NWs occurred almost at equimolar concentration (Fig. S2†). The morphology and size of the resulting S2P–CeO_2_–ASOs nanoplatform were further confirmed with TEM (Fig. 1B). Electrophoretic gel analysis showed that S2P–CeO_2_–ASOs, when compared to free ASOs, were resistant to 10 μg mL^–1^ DNase I treatment and highly stable in 10% FBS supplemented DMEM at 37 °C for 24 h. Importantly, S2P–CeO_2_–ASOs maintained a high ASO loading upon pH change (Fig. 1C). This is mainly attributed to the steric blocking effect of the PEG layer^31^ and the stable nature of the Ce^4+^–phosphate oxygen coordination conjunction,^33^ which reduced enzymatic degradation and unexpected desorption of the ASOs. Because of the stronger binding ability between Ce^4+^ and peroxo, approximately 90% of the loaded ASOs was released within 5 min in the presence of 100 μM H_2_O_2_ (Fig. S3†). The amount of ASOs released was proportional to the H_2_O_2_ concentration (Fig. 1D). Further inspection of the ASOs release caused by other components, such as superoxide anions (O_2_˙^–^), hydroxyl radicals (˙OH), cysteine (Cys), glutathione (GSH), ascorbic acid (AA), and adenosine triphosphate (ATP), possibly coexisting with H_2_O_2_, revealed that these species did not cause a remarkable release of ASOs (Fig. S3†). These results together indicated that S2P–CeO_2_–ASOs formed a stable uniform-sized nanoplatform capable of efficient loading and H_2_O_2_-specific controlled release of mTOR ASOs.

### *In vitro* plaque-targeting ability and mTOR silencing efficacy of S2P–CeO_2_–ASOs

**Fig. 2:**
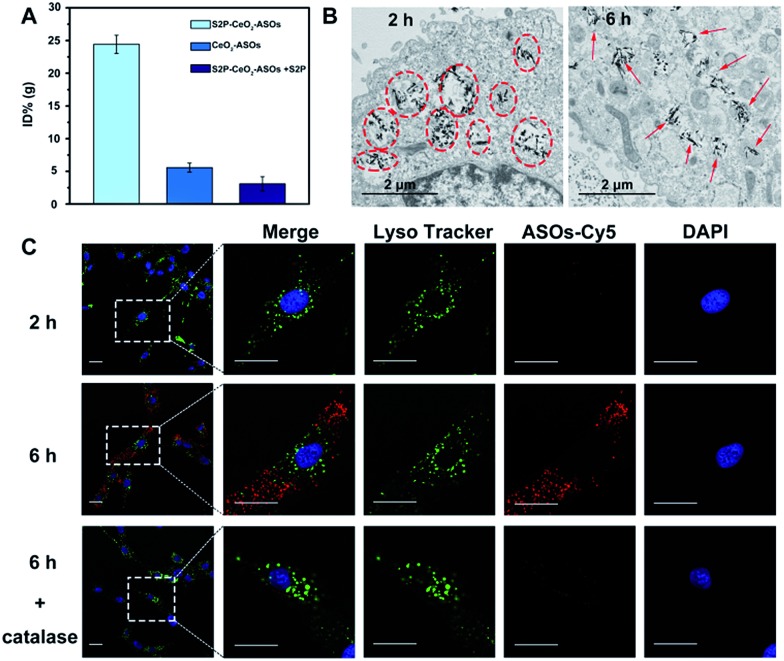
*In vitro* plaque-targeting and endosomal escape ability of S2P–CeO_2_–ASOs. (A) ICP-AES analysis of VSMCs incubated with S2P–CeO_2_–ASOs (50 μg mL^–1^), CeO_2_–ASOs (50 μg mL^–1^) and S2P–CeO_2_–ASOs (50 μg mL^–1^) with S2P peptide (1 mg mL^–1^) at 37 °C for 6 h. (B) TEM images of VSMCs incubated with 50 μg mL^–1^ S2P–CeO_2_–ASOs for 2 h and 6 h. The circles highlight S2P–CeO_2_–ASOs taken up into endosomes or lysosomes. The arrows indicate S2P–CeO_2_–ASOs released into the cytoplasm. Scale bar = 2 μm. (C) Fluorescence images of VSMCs incubated with Cy5–ASOs loaded S2P–CeO_2_ at 37 °C for 2 h and 6 h. Endosomes are stained by Lysotracker (green) and nuclei were stained with DAPI (blue). Catalase (100 μM) was used as a H_2_O_2_ scavenger. The white dashed square is the area of the magnification shown on the right (scale bar = 25 μm).

After acquiring the nanoplatform with a high loading efficiency and H_2_O_2_-responsive release property, an inductive coupling plasma atomic emission spectrometer (ICP-AES, based on a total Ce content uptaken per cell) was employed to evaluate its *in vitro* plaque-targeting ability. The uptake of the S2P–CeO_2_–ASOs nanoplatform was facilitated through a stabilin-2-mediated endocytosis pathway. Stabilin-2 is a transmembrane protein expressed predominantly in VSMCs of atherosclerotic plaques (Fig. S4†), which can recognize and bind S2P peptides on the nanoplatform. As shown in Fig. S5,† VSMCs incubated with S2P–CeO_2_–ASOs showed a significant uptake starting at 2 h and saturating at 6 h. This uptake was concentration-dependent with a maximum Ce content per cell at 50 μg mL^–1^ (Fig. S6†). Importantly, VSMCs uptake of S2P–CeO_2_–ASOs was significantly higher than that of S2P absent CeO_2_–ASOs and S2P–CeO_2_–ASOs with a blocking dose (1 mg mL^–1^) of S2P peptide (Fig. 2A). In addition, the S2P–CeO_2_–ASOs was not taken up efficiently by any of the stabilin-2-negative cell lines (Fig. S7†). This targeted uptake by cultured VSMCs demonstrated *in vitro* that S2P–CeO_2_–ASOs have a specific and strong binding affinity with stabilin-2 and therefore can target and penetrate stabilin-2-overexpressing VSMCs in the plaque area.

Next, the endosomal escape ability of S2P–CeO_2_–ASOs was examined using TEM. The piercing of the endolysosomal membrane by high aspect ratio CeO_2_ cores was seen, which led to the release of the S2P–CeO_2_–ASOs nanoplatform into the cytoplasm after 6 h of incubation (indicated by the red arrows in Fig. 2B). An MTT assay (Fig. S8†) showed that localized damage inflicted on the endosomal membrane did not induce cell death. Red fluorescently (Cy5) tagged ASOs were further used to confirm the endosomal escape and cytosolic H_2_O_2_-triggered release. As shown in Fig. 2C, owing to the high quenching ability of CeO_2_ NWs and excellent pH stability (5–8) of CeO_2_ NW–DNA coordination complexes,^36^ there was no red fluorescence signal when S2P–CeO_2_–ASOs nanoplatforms were localized inside the endosomes/lysosomes at the early time point (2 h). When the nanoplatforms entered the cytoplasm (6 h), the presence of H_2_O_2_ (Fig. S9†) induced liberation of the absorbed ASOs from the CeO_2_ surface as a result of the stronger binding ability between peroxo and Ce^4+^ in comparison to that between phosphate oxygen and Ce^4+^. This resulted in a dramatic increase in the red fluorescence signal. The lack of colocalization with LysoTracker staining indicated the effective endosomal escape of S2P–CeO_2_–ASOs. However, upon addition of a H_2_O_2_ scavenger (catalase, 100 μM), a marked decrease in the red fluorescence intensity was observed, further confirming that the nanoplatform allowed for “on-demand” release of ASOs in response to cytosolic H_2_O_2_.

**Fig. 3:**
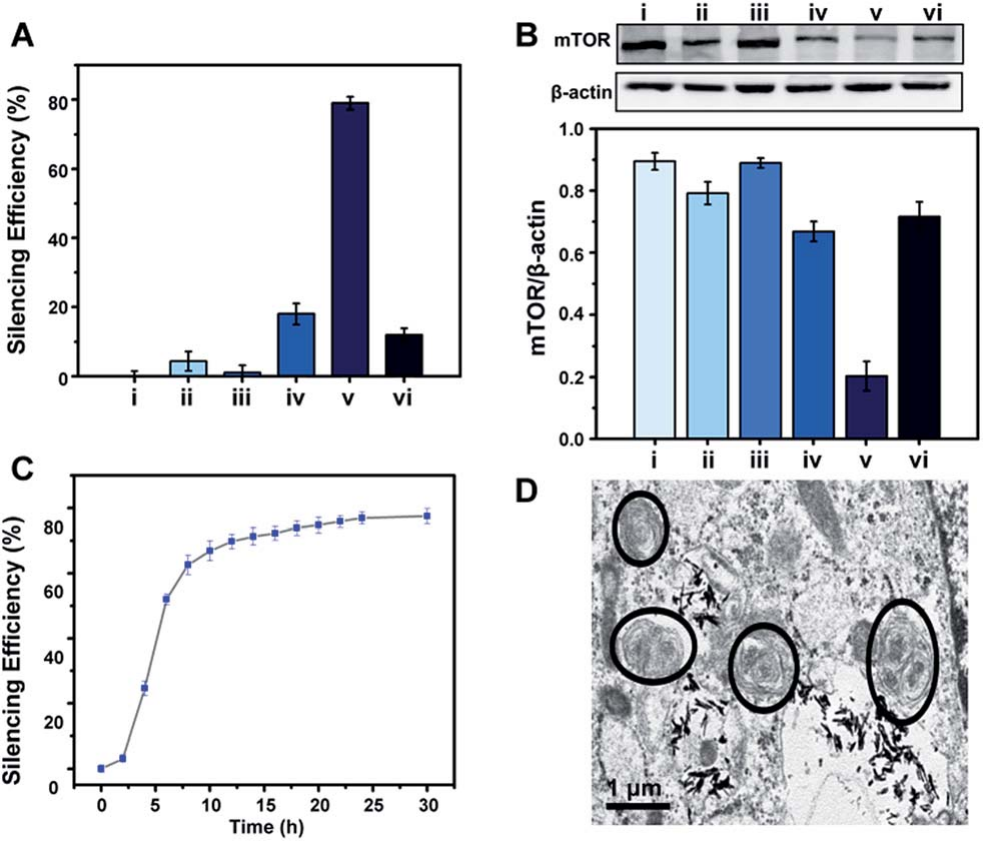
*In vitro* gene silencing efficacy of S2P–CeO_2_–ASOs in VSMCs. (A) qRT-PCR and (B) western blot analysis of mTOR expression in VSMCs: (i) blank, and treated with (ii) free ASOs, (iii) S2P–CeO_2_, (iv) CeO_2_–ASOs, (v) S2P–CeO_2_–ASOs and (vi) S2P–CeO_2_–ASOs + catalase for 24 h (ASO dose: 80 ng; catalase: 100 μM). (C) Dependence of the mTOR silencing percentage upon incubation time after treating VSMCs with S2P–CeO_2_–ASOs. (D) Representative TEM images of autophagosomes after treatment with S2P–CeO_2_–ASOs for 24 h. The black circles outline the double-membrane structures of the autophagosomes. Scale bar = 1 μm.

To demonstrate the feasibility of the S2P–CeO_2_–ASOs nanoplatform toward enhanced mTOR gene silencing, various controls and time-silencing dependence studies were carried out to investigate and optimize the *in vitro* intracellular mRNA silencing mechanism involved in the mTOR knockdown. Fig. 3A shows the qRT-PCR results of VSMCs after they have been exposed to different conditions and incubated for 24 h in the culture medium. mTOR gene-silencing achieved 79% with S2P–CeO_2_–ASOs at an 80 ng ASO concentration, as compared to 18% achieved with CeO_2_–ASOs (Fig. 3A, bar v *vs.* bar iv), which was ∼4 times lower than S2P-conjugated silencing abilities. It has also been demonstrated that S2P–CeO_2_–ASOs better facilitate the ASO internalization process, as the nanoplatform offers an 18-fold enhanced silencing when compared to free ASOs in solution at the same concentration (Fig. 3A, bar v *vs.* bar ii). Furthermore, to ensure that the silencing efficiency was a result of the H_2_O_2_-triggered release of ASOs, cells treated with catalase and S2P–CeO_2_–ASOs and cells treated with S2P–CeO_2_ cores lacking the ASOs (Fig. 3A, bar vi and bar iii) were also analyzed. These control experiments resulted in negligible mTOR downregulation. In addition, the S2P–CeO_2_–ASOs showed a fast silencing response (52% after a 6 h treatment), reaching a silencing percentage plateau 24 h post-treatment (Fig. 3C). Western blots (Fig. 3B) and immunofluorescence analysis (Fig. S10 and S11†) further confirmed that the mTOR protein levels were greatly reduced by the nanoplatform at 24 h. In view of these results, there are clear advantages in the efficiency and speed of the mTOR gene knockdown with the S2P–CeO_2_–ASOs. With the suppressed mTOR expression, the autophagy, measured as the ratio of LC3II/LC3I (Fig. S12†), and the number of double-membrane autophagosomes (Fig. 3D) increased significantly after the treatment with S2P–CeO_2_–ASOs for 24 h. The S2P–CeO_2_–ASOs-induced autophagy activation resembled the activation achieved by the direct treatment of cells with rapamycin. Consequently, activation of the autophagy inhibited oxidized low-density lipoprotein (oxLDL)-induced VSMC foam cell formation, which was manifested by a decreased lipid droplet accumulation (Fig. S13A†) and total cholesterol level (Fig. S13B†). This decrease also occurred in the rapamycin treated group.

### Pharmacokinetic and *in vivo* plaque-targeting ability of S2P–CeO_2_–ASOs

**Fig. 4:**
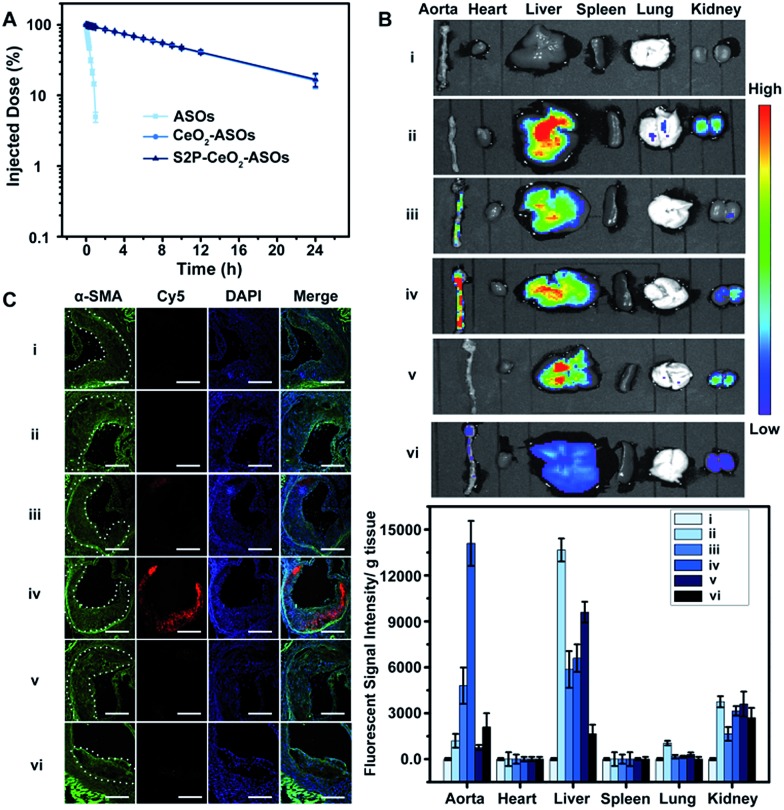
The pharmacokinetic and *in vivo* plaque-targeting ability of S2P–CeO_2_–ASOs. (A) Pharmacokinetics of free ASOs, CeO_2_–ASOs and S2P–CeO_2_–ASOs (ASO dose: 0.5 mg kg^–1^). Data are shown as the mean ± S.D. (*n* = 3). (B) Fluorescence images of the aortas and main organs of the plaque-bearing ApoE^–^/^–^ mice sacrificed 24 h postinjection of (i) PBS, (ii) free ASOs, (iii) CeO_2_–ASOs, (iv) S2P–CeO_2_–ASOs, (v) S2P peptide followed by S2P–CeO_2_–ASOs and (vi) S2P–CeO_2_–ASOs with H_2_O_2_ scavenger catalase (ASO dose: 0.5 mg kg^–1^; catalase: 1 mg kg^–1^). Lower panels: quantification of the fluorescence intensity in the tissue. Data are shown as the mean ± S.D. (*n* = 3). (C) Immunofluorescence micrographs of aortic arch cross-sections of groups (i), (ii), (iii), (iv), (v) and (vi) indicated in (B). From the left, α-SMA (green, representing VSMC), Cy5 (red, representing S2P–CeO_2_–ASOs or the controls), DAPI (blue) and the merged image. The dashed line indicates the plaque border. Scale bar = 100 μm.

Having established that S2P–CeO_2_–ASOs can activate autophagy and inhibit VSMC foam cell formation through mTOR gene expression silencing, we assessed their pharmacokinetics and *in vivo* plaque-targeting ability. The pharmacokinetics of S2P–CeO_2_–ASOs was examined by intravenous injection of Cy5-tagged ASOs loaded nanoplatforms to healthy ApoE^–^/^–^ mice (ASO dose: 0.5 mg kg^–1^, *n* = 3). As shown in Fig. 4A, the blood half-life (*t*_1/2_) of S2P–CeO_2_–ASOs was around 9.33 h, which was comparable to the *t*_1/2_ of CeO_2_–ASOs (∼9.16 h), but far longer than that of free ASOs (*t*_1/2_ < 20 min). This prolonged half-life in the blood circulation was mainly due to the protection of the PEG outer layer and stable nature of the Ce^4+^–phosphate oxygen coordination conjunction. The *in vivo* plaque-targeting ability of S2P–CeO_2_–ASOs was assessed by intravenously injecting Cy5-tagged ASOs loaded nanoplatforms into plaque-bearing ApoE^–^/^–^ mice (ASO dose: 0.5 mg kg^–1^, *n* = 3). Fig. 4B showed the fluorescence images of the aorta and major organs excised 24 h post-injection. With S2P-mediated stabilin-2 (overexpression on plaque, Fig. S14†) targeting and H_2_O_2_ (overgeneration in plaque, Fig. S15†) triggered release of ASOs, S2P–CeO_2_–ASOs showed a high accumulation in the aorta corresponding to the bright fluorescence. When administration of S2P peptide (1 mg kg^–1^) was performed 1 h before S2P–CeO_2_–ASOs injection, stabilin-2 blocking significantly reduced the aorta accumulation of S2P–CeO_2_–ASOs. In comparison, CeO_2_–ASOs or free ASOs presented a high accumulation in the liver and kidneys and an extremely low accumulation in the aorta. Fig. 4B also showed a marked decrease in the fluorescence intensity in mice treated with S2P–CeO_2_–ASOs with H_2_O_2_ scavenger catalase (1 mg kg^–1^), further confirming the ability of the nanoplatform to release ASOs “on-demand” in response to H_2_O_2_.

It has been identified that the blood vessels surrounding atherosclerotic plaques are defective and immature.^40^,^41^ Once a large proportion of S2P–CeO_2_–ASOs accumulated in the aorta, the leaky vasculature allows them to penetrate and be retained in the plaques. To evaluate the plaque-penetrating ability of the S2P–CeO_2_–ASOs, aortic arches were isolated from mice and their localization was determined by an immunohistochemistry study. Immunofluorescence micrographs of aortic arch cross-sections (Fig. 4C) clearly showed that S2P–CeO_2_–ASOs can (shown with the red fluorescence of Cy5) easily pass through the arterial wall and become trapped by VSMC in atherosclerotic plaques (shown with the green fluorescence of α-SMA) *via* S2P mediated stabilin-2 targeting. As expected, non-coated CeO_2_–ASOs and free ASOs did not show plaque-specific targeting. These results strongly demonstrated the deep plaque-penetrating and H_2_O_2_-triggered release characteristics of S2P–CeO_2_–ASOs.

### *In vivo* mTOR silencing and anti-atherosclerosis efficacy of S2P–CeO_2_–ASOs

**Fig. 5:**
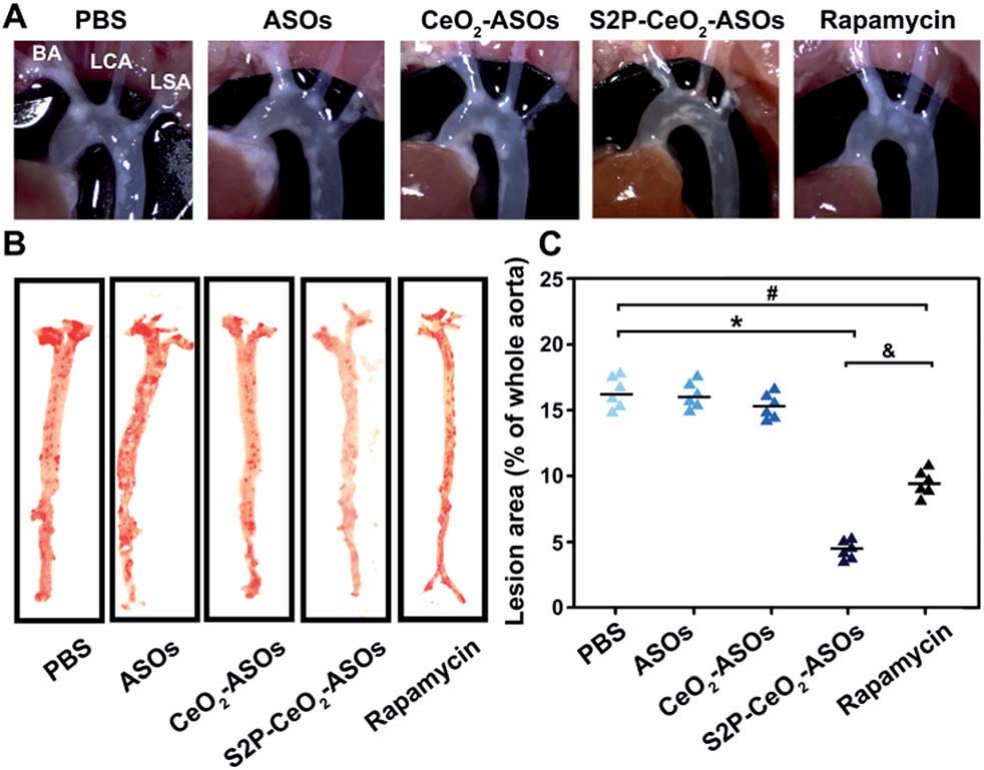
The *in vivo* anti-atherosclerosis efficacy of S2P–CeO_2_–ASOs. (A) *In situ* aortic arch lesions in ApoE^–^/^–^ mice on a high-fat diet after treatment by (i) PBS, (ii) free ASOs, (iii) CeO_2_–ASOs, (iv) S2P–CeO_2_–ASOs and (v) rapamycin (ASO dose: 0.5 mg kg^–1^; rapamycin: 4 mg kg^–1^). Representative images of the plaque at the origins of the brachiocephalic artery (BA), left carotid artery (LCA) and left subclavian artery (LSA). (B) *En face* aortic lesions in the (i)–(v) groups. Representative images of atherosclerotic lesions in oil red-O-stained aorta. (C) Quantification of the stained area as a percentage of the whole aorta. Data are shown as the mean ± S.D. (*n* = 6). *P < 0.05 for S2P–CeO_2_–ASOs *vs.* PBS, ^#^*P* < 0.05 for rapamycin *vs.* PBS, ^&^*P* < 0.05 for S2P–CeO_2_–ASOs *vs.* rapamycin.

To examine the inhibition of the mTOR expression in aortic atherosclerotic lesions, S2P–CeO_2_–ASOs nanoplatforms were intravenously administered into ApoE^–^/^–^ mice fed with a high-fat diet (ASO dose: 0.5 mg kg^–1^, *n* = 3). Aortas were harvested three days post-injection and the *in vivo* mTOR expression was evaluated by western blot analysis. By virtue of plaque-penetration, S2P–CeO_2_–ASOs induced >75% knockdown of the mTOR expression in the aortas. In contrast, there was <26% knockdown for the CeO_2_–ASOs and free ASOs controls (Fig. S16A†). As a result, significant autophagy activation was observed in mice treated with S2P–CeO_2_–ASOs, as evidenced by the increased LC3II/LC3I ratio in the aortic lesions (Fig. S16B†). To evaluate whether mTOR silencing had an anti-atherosclerosis effect, the S2P–CeO_2_–ASOs nanoplatforms were intravenously injected into the ApoE^–^/^–^ mice twice a week at a dose of 0.5 mg kg^–1^ ASOs (*n* = 6). After 12 weeks, the S2P–CeO_2_–ASOs treatment had the best therapeutic results in limiting atherosclerotic lesion progression in the ApoE^–^/^–^ mice on a high-fat diet compared to the controls. Examination of the aortic arch with a stereo dissecting microscope (Fig. 5A) showed a marked decrease in the progression of atherogenesis, and *en face* preparations (Fig. 5B and C) revealed that the oil red-O-stained area was also reduced by 67.1%. In comparison, a smaller decrease in the progression of atherosclerotic lesions (41.3%) was observed in the rapamycin group. The main reason for this is that rapamycin is water insoluble and lacks specificity. Consequently, it has a low bioavailability and cannot confine its therapeutic effects to atherosclerotic regions as seen with the S2P–CeO_2_–ASOs treatment. Notably, S2P–CeO_2_–ASOs showed good biocompatibility, as evidenced by the lack of histological changes in tissues of the heart, liver, spleen, lung, or kidneys and no obvious influence on the mouse body weight (Fig. S17 and S18†).

## Conclusions

In summary, we have successfully developed a H_2_O_2_-responsive and plaque-penetrating S2P–CeO_2_–ASOs nanoplatform for targeted mTOR ASOs delivery. *In vitro* and *in vivo* results demonstrated that this nanoplatform has a long blood circulation time, and can efficiently pass through the arterial wall, penetrating into the plaque through specific recognition between S2P ligands and overexpressed stabilin-2 on VSMCs. With the high aspect ratio CeO_2_ core and H_2_O_2_-based competitive coordination, this nanoplatform can effectively escape from endosomes and release therapeutic ASOs “on-demand” into the cytoplasm, leading to a significant suppression of the mTOR expression and atherosclerotic plaque formation in ApoE^–^/^–^ mice fed with a high-fat diet. After 12 weeks of treatment, the nanoplatform showed a more effective therapeutic outcome compared to mTOR inhibitor rapamycin. Accordingly, this targeted gene-silencing nanoplatform opens up new opportunities for developing CeO_2_-based nanotheranostics and possibly a robust atherosclerosis treatment strategy for clinical applications.

## Conflicts of interest

There are no conflicts to declare.

## Supplementary Material

Supplementary information
