# Supplementary material for: H2O2-responsive and plaque-penetrating nanoplatform for mTOR gene silencing with robust anti-atherosclerosis efficacy
Source: Chem Sci. 2017 Oct 27;9(2):439–45. doi: 10.1039/c7sc03582a (PMC5868311; doi:10.1039/c7sc03582a)
Supplement: Supplementary file 1 [file SC-009-C7SC03582A-s001.pdf]

Electronic Supplementary Material (ESI) for Chemical Science.
This journal is © The Royal Society of Chemistry 2017

Electronic Supplementary Information

H_2O_2-responsive and plaque-penetrating nanoplatform for mTOR gene silencing with robust anti-atherosclerosis efficacy

Wen Gao,‡[a] Yujie Zhao,‡[a] Xiang Li,[a] Yuhui Sun,[a] Michelle Cai,[b] Wenhua Cao,[a] Zhenhua Liu,[a] Lili Tong,[a] Guanwei Cui[a] and Bo Tang*[a]

[a]College of Chemistry, Chemical Engineering and Materials Science, Collaborative Innovation Center of Functionalized Probes for Chemical Imaging in Universities of Shandong, Key Laboratory of Molecular and Nano Probes, Ministry of Education, Institute of Biomedical Sciences, Shandong Normal University, Jinan 250014, P.R. China.

[b]Faculty of Science, Western University, London, Ontario N6A5B7, Canada.

‡These authors contributed equally to this work.

Corresponding Authors

*E-mail: tangb@sdnu.edu.cn. Fax: +86 531 86180017

Contents:

Part A: Experimental Methods;

Part B: Supplementary tables and figures (S1-S16).

Part A: Experimental Methods

Materials. All chemicals and solvents used were of analytical grade or above. Cerium(III) nitrate (99.5%, $Ce(NO_3)_3 \cdot 6H_2O$) were purchased from Shanghai Siyu Chemical Technology Co. Ltd. (Shanghai, China). Sodium hydroxide (NaOH), Sodium hydroxide (NaCl), tris(2-carboxyethyl)phosphine hydrochloride (TCEP), 4-(2-hydroxyethyl)-1-piperazineethanesulfonic acid (HEPES), Sodium dodecylsulfate (SDS) were obtained from Sinopharm Chemical Reagent. Co. Ltd. (Shanghai, China). 3-(4,5-dimethyl-thiazol-2-yl)-2,5-diphenyltetrazolium bromide (MTT) and rapamycin were purchased from Sigma-Aldrich (St. Louis, MO, USA). Antibody against LC3, polyvinylidene fluoride (PVDF) membrane, the enhanced chemiluminescence (ECL) substrate, Lyso-Tracker green and DAPI were purchased from Beyotime Inst. Biotech, (Haimen, China). Antibody against mTOR, Stabilin-2 and β-actin were purchased from Abcam (Burlingame, CA, USA). S2P peptide (CRTLTVRKC) was purchased from GLS Biochem Systems, Inc (Shanghai, China). Deoxyribonuclease I (DNase I) was purchased from Solarbio (Beijing, China). Maleimide-poly(ethylene glycol)-amine (Mal-PEG-NH_2; molecular weight of PEG: 2000) was purchased from Jenkem Technology Co., Ltd. (Beijing, China). Cell culture products, unless mentioned otherwise were purchased from Gibco, Invitrogen. All DNA oligonucleotides were synthesized and purified by Sangon Biotechnology Co., Ltd. (Shanghai, China) and their sequences were listed in Table S1 and S3. Water was purified with a Sartorius Arium 611 VF system (Sartorius AG, Germany) to a resistivity of 18.2 MΩ·cm.

Instruments. High resolution transmission electron microscopy (HRTEM) was carried out on a JEM-2100 electron microscope. Zeta potential was measured with a Malvern Zeta Sizer Nano (Malvern Instruments). X-ray diffraction (XRD) analysis was carried out on a D/Max 2500 V/PC X-ray diffractometer using Cu (40 kV, 30 mA) radiation. All pH measurements were performed with a pH-3c digital pH-meter (Shanghai LeiCi Device Works, Shanghai, China) with a combined glasscalomel electrode. Nanodrop experiment was performed with a NanoDrop 2000 Spectrophotometer (Thermo Scientific).

Absorbance in MTT assay was measured in a microplate reader (RT 6000, Rayto, USA). Confocal fluorescence imaging studies were performed with a TCS SP8 confocal laser scanning microscopy (Leica Co., Ltd. Germany) with an objective lens (×63). Quantitative real time (qRT)-PCR was carried out on LineGene 9620 sequence detection system (Bioer, Hangzhou, China). In vivo imaging was performed with Caliper IVIS Lumina III (Caliper Co., USA).

Preparation of CeO_2 and S2P-CeO_2-ASOs. CeO_2 NWs were prepared using a modified hydrothermal method.[1] Briefly, 5mL of 0.05 M $Ce(NO_3)_3$ solution was added dropwise into 35 mL of 6 M NaOH solution. The mixture was aged at room temperature under continuous stirring for 30 min. The white slurry was then transferred into an autoclave and maintained at 100 °C for 24 h to get highly crystallized CeO_2 NWs with a high aspect ratio. After cooling to room temperature, the sediment was washed by deionized water and ethanol alternately for three times and then dried at 60 ºC overnight.

To prepare the S2P-CeO_2-ASOs nanoplatform, CeO_2 NWs was first modified with Mal-PEG-NH_2 (MW 2000) by stirring the NPs (10 mg) with the Mal-PEG-NH_2 (2 mg) in ethanol solution for 4 h at room temperature. Then, PEG-capped CeO_2 NWs were pelleted down, washed, and resuspended in HEPES (pH 7.4). Next, 10 mg of as-prepared PEG-CeO_2 NWs were reacted with 1.5 mg of S2P peptides (CRTLTVRKC) in 5 mL of HEPES (pH 8.0) for 12 h at room temperature in the presence of TCEP. Excess S2P peptide were then removed by filtration (MWCO =1000 kDa). S2P-PEGylated CeO_2 NW was obtained by coupling the thiol groups of S2P peptide with the maleimide groups on PEG. Finally, loading of siRNA was achieved by mixing S2P-PEGylated CeO_2 NWs (10 mg) and ASO1 and ASO2 (10 nM) in 10 mM HEPES (pH 7.4) with 150 mM concentrations of NaCl (1 h, 37 °C, 200 rpm shaking). Free ASOs were removed by filtration (MWCO =100 kDa). The final nanoplatforms were washed and centrifuged several times with DNase/RNAase free water and diluted to a known volume, estimated for their particle size, Zeta potential and the loaded ASOs level, prior to use in different

experiments.

Stability analysis. S2P-CeO_2-ASOs (50 μg/mL) were incubated with DNase I (1, 10 μg/mL), 10% FBS supplemented DMEM medium or HEPES buffer (pH 5.0-8.0) at 37 °C After incubation for specific time points, the integrity of the S2P-CeO_2-ASOs nanoplatform was examined through native PAGE gel electrophoresis. Free ASOs (80 ng) was used as negative control.

H_2O_2-triggered release of ASOs. 50 μg of S2P-CeO_2-ASOs was incubated with 1 mM of H_2O_2 in 1.0 mL of HEPES buffer (10 mM, pH 7.4, 150 mM NaCl) for 30 min at 37°C. Other components possibly coexisting with H_2O_2 such as superoxide anion ($O_2^{\cdot -}$), hydroxyl radical ($^{\bullet}OH$), cysteine (Cys), glutathione (GSH) ascorbic acid (AA), adenosine triphosphate (ATP) were added to the control experiments, respectively. The release of ASOs from the nanoplatform was also studied as a function of the amount of H_2O_2 (0, 0.01, 0.02, 0.05, 0.1, 0.2, 0.5, 1 mM; 30 min at 37 °C). ASOs release was monitored by NanoDrop spectrophotometer at appropriate time intervals.

Cell culture. Human umbilical vein endothelial cell line (HUVEC) and human embryonic kidney 293 cell line (HEK293) were purchased from the Committee on Type Culture Collection of the Chinese Academy of Sciences. Rat aortic vascular smooth muscle cells (VSMCs) were obtained from Dingguo Changsheng Biotechnology Co., Ltd. (Beijing, China). All types of cells were cultured in Dulbecco's modified Eagles medium (DMEM) with 10% fetal bovine serum and 100 U/mL of 1% antibiotics penicillin/streptomycin and maintained at 37 °C in a 5% CO_2/95% air humidified incubator (MCO-15AC, SANYO). To ensure maintenance of the contractile phenotype, we used cells in the second to eighth passages for each experiment.

Cellular Uptake. VSMCs were seeded in 6-well plates at 5×10^4 cells/well 24 h prior to addition of the test substance. Then, sterilized S2P-CeO_2-ASOs or CeO_2-ASOs was cultured with VSMCs at a concentration of (50 μg/mL). In blocking group, excess amount of S2P peptide (1 mg/mL) was added to cells 30 min before S2P-CeO_2-ASOs.

For quantification of nanoparticles uptaken in VSMCs, ICP-AES measurements were performed. After 4 h incubation, the media was aspirated off, cells were washed twice with PBS and digested with 70% w/v nitric acid containing 10 ppm Yttrium at 80 ºC for 6 h. After cooling to room temperature, condensates were collected by centrifugation, and diluted to 5 mL with water. Samples were then filtered through a 0.45 μm hydrophilic PVDF membrane into 15 mL Falcon tubes. Standards were prepared by diluting 1000 ppm cerium standards solution to 100 ppm, 10 ppm, 1 ppm, 0.2 ppm, 0.04 ppm with 15% w/v nitric acid. ICP-AES was performed on the samples using the Optima 7300 (Perkin Elmer). Another batch of VSMCs was exposed to different concentrations (0-60 μg/mL) of S2P-CeO_2-ASOs, and the internalization of NPs were monitored at specific time intervals. Two control cells, HUVEC[2] and HEK293,[3] were also introduced to better demonstrated the stabilin-2 specificity of S2P-CeO_2-ASOs.

Endosomal escape. VSMCs were seeded at an initial density of 5×10^4 cells/dish in 6-well plates or 20-mm glass bottom dishes and incubated for 24 h before adding the test substance. The endosomal escape of S2P-CeO_2-ASOs was visualized by transmission electron microscopy (TEM) and confocal microscopy. (1) TEM measurements. Fresh medium containing sterilized S2P-CeO_2-ASOs (50 μg/mL) were added, and the cells were incubated for 2 h and 6 h at 37 °C, respectively. After that, cells were trypsinized, centrifuged, and fixed in 2.5% glutaraldehyde in sodium cacodylate buffer (pH 7.4， 0.1 M) for 1 h at room temperature and rinsed. Cells were then post fixed 1 h in 2% osmium tetroxide with 3% potassium ferrocyanide and rinsed, next enbloc staining with a 2% aqueous uranyl acetate solution and dehydration through a graded series of alcohol (50%, 70%, 80%, 95%, 100%). Then they were put into two changes of propylene oxide, a series of propylene/epon dilutions and embedded in 100% Epon. The 70 nm thin sections were cut on a Leica UC6 ultramicrotome, and images were taken on a JEOL 1200 EX (JEOL, Ltd. Tokyo, Japan) using an AMT 2k digital Camera. (2) Confocal imaging. Fresh medium containing sterilized Cy5-ASOs loaded S2P-CeO_2 (50 μg/mL) were added,

and the cells were incubated for 2 h and 6 h at 37 °C, respectively. Catalase (100 μM) was used as a H_2O_2 scavenger. After removing the medium and subsequently washing with PBS buffer, the endosomes and nuclei were stained with Lysotracker green and DAPI, respectively. The cells were then viewed under TCS SP8 confocal laser scanning microscopy (Leica Co., Ltd. Germany) with 405 nm, 488 nm and 563 nm excitation.

Cytotoxicity assay. VSMCs were seeded in 96-well plates (10^4 cells/well) and cultured overnight. Then fresh medium containing sterilized S2P-CeO_2-ASOs (50 μg/mL) was added to each well and incubated for 2, 6, 12 and 24 h, respectively. Cell viability was evaluated by MTT assay according to the previously reported method.[4] Cells incubated with free medium served as a negative control.

***In vitro* silencing efficacy.** VSMCs were seeded in 6-well cell plates or glass slides (10^6 cells) and cultured overnight. Then fresh medium containing sterilized (i) blank (ii) free ASOs, (iii) S2P-CeO_2, (iv) CeO_2-ASOs, (v) S2P-CeO_2-ASOs and (vi) S2P-CeO_2-ASOs + catalase was respectively added to each well at 80 ng ASOs dose. After incubation for predetermined time intervals, mRNA and protein expression levels of mTOR in VSMCs were determined by quantitative-real time-RT-PCR (qRT-PCR), western blotting and immunofluorescence assay. (1) qRT-PCR. Total RNA was extracted from each group of cells using an RNAeasy™ kit (Beyotime Inst. Biotech, Haimen, China), according to the manufacturer's instructions, followed by reverse-transcription using Superscript IV reverse transcriptase (Invitrogen). qRT-PCR was performed on the LineGene 9620 sequence detection system (Bioer, Hangzhou, China) using QuantiNova SYBR Green PCR Kit (Qiagen, Valencia, CA, USA). Primers used for qRT-PCR are listed in the supporting information (Table S3). The expression of mTOR was analyzed using GAPDH as the internal reference. (2) Western blotting. Each group of cells was lysed in RIPA buffer supplemented with cocktail protease inhibitors and PMSF (1 mM), respectively. The amount of protein was measured using a protein assay kit (Beyotime, China). Equal amounts of protein (50 μg) were resolved on 6% SDS-PAGE gels and

electro-blotted onto a polyvinylidene fluoride (PVDF) membrane and blocked with TBS containing 0.05% Tween-20 and 5% nonfat milk powder for 1 h. Next, membranes were incubated overnight with primary antibodies against mTOR (1:1,000) and β-actin (1:1,000), followed by HRP-conjugated secondary antibody (1:50,000) for 1 h. Detection was carried out by incubating membranes for 5 min with the enhanced chemiluminescence reagent, followed by exposure to ChemiDoc™ Touch Imaging system (Bio-Rad, Hercules, CA, USA). (3) Immunofluorescence. Each group of cells was fixed with 4% paraformaldehyde for 20 minutes at 4 ºC, respectively. Nonspecific proteins were blocked with goat serum blocking solution for 1 h at 37 ºC. Then primary antibody for mTOR (1:400) was added and incubated overnight at 4 ºC. After washing the cells with PBS for 3 times, FITC-labeled secondary antibody (1:200) was added and incubated in the dark for another 1 h at 37 ºC. Finally, the nucleus was counterstained with DAPI (10 mg/mL) for 5 min, washed with PBS and placed into serial diluted glycerol solution from 100% to 50% for confocal microscopy observation.

Autophagy assay. VSMCs were seeded in 6-well cell plates or glass slides (10^6 cells) and cultured overnight. Then fresh medium containing sterilized (i) blank (ii) free ASOs, (iii) S2P-CeO_2, (iv) CeO_2-ASOs, (v) S2P-CeO_2-ASOs and (vi) S2P-CeO_2-ASOs + catalase was respectively added to each well at 80 ng ASOs dose. Fresh medium containing (vii) rapamycin (10 nM) were used as a positive control. After incubation for 24 h, autophagy activation in VSMCs was determined by TEM and western blotting. (1) Characterization of autophagosomes. The above-mentioned seven groups of cells were collected respectively, and fixed in 2.5% glutaraldehyde in sodium cacodylate buffer (pH 7.4，0.1 M) for 1 h at room temperature. The accumulation of typical autophagosomes with double membranes was identified morphologically by TEM following the same procedure as described above. (2) Western blot analysis of LC3II/LC3I ratio. The above-mentioned seven groups of cells were collected and lysed, respectively. Changes in LC3II and LC3I expression were analyzed by western blotting following the same procedure as

described above (primary antibody against LC3B: 1:1,000).

Foam cell formation assay. Foam cells were quantified by Oil Red O staining and intracellular total cholesterol content[5]. (1) Oil Red O staining. VSMCs were seeded in 20-mm glass bottom dishes (10^6 cells) and cultured overnight. After pretreatment with oxLDL (60 μg/mL) for 24 h, VSMCs were divided into the above-mentioned seven groups. After 24 h treatment, VSMCs were fixed with 4% paraformaldehyde, washed with 60% isopropanol, and stained for lipid droplets with Oil Red O in 60% isopropanol. Foam cells were photographed under a fluorescence microscope with a ×40 objective lens (Leica DMI3000B, Germany). (2) Total Cholesterol Assay. Intracellular total cholesterol content was determined by enzymatic assay (Applygen Technologies, Beijing, China). After treatment under the same conditions, VSMCs were collected and washed three times with PBS. Isopropylalcohol (100 μM) was used to extract the intracellular lipids by ultrasonication. After centrifugation for 5 min at 1,500 *g*, the supernatant was used to determine total cholesterol. The total cellular protein levels were measured using a protein assay kit (Beyotime, China). The results were expressed in μg of cholesterol per mg of cellular protein.

Animals. All animal experiments were carried out according to the guidelines of the Institutional Animal Care and Use Committee (People's Republic of China). $ApoE^{-/-}$ mice (6-8 weeks old, male) were purchased from Changzhou Cavens Laboratory Animal Co. Ltd. (Changzhou, China) and housed under controlled conditions (12/12 h light/dark cycle; humidity 50-60%; 24-25 °C room temperature), with free access to water and chow. To induce the development of atherosclerotic lesions, mice were fed with the high-fat diet (HFD, 20% fat, 20% sugar, and 1.25% cholesterol) for 12 weeks.

Pharmacokinetics study. Healthy $ApoE^{-/-}$ mice were randomly divided into three groups (n = 3) and given an intravenous injection of (i) free Cy5-labelled ASOs, (ii) Cy5-labelled CeO_2-ASOs, (iii) Cy5-labelled S2P-CeO_2-ASOs at 0.5 mg/kg ASOs dose. At predetermined time intervals, 20 μL of tail vein blood was withdrawn using a tube

containing heparin, and the wound was pressed for several seconds to stop the bleeding. For group (ii) and (iii), 1mM H_2O_2 was added to release the loaded Cy5-labelled ASOs. Then, fluorescence intensity of Cy5-labelled ASOs in the blood was determined by an RT 6000 microplate reader. The blood circulation half-life ($t_{1/2}$) was calculated from a first-order fit of the data.

Biodistribution study. Plaque-bearing ApoE$^{-/-}$ mice were randomly divided into four groups (n = 3) and given an intravenous injection of (i) PBS, (ii) free Cy5-labelled ASOs, (iii) Cy5-labelled CeO_2-ASOs, and (iv) Cy5-labelled S2P-CeO_2-ASOs at 0.5 mg/kg ASOs dose. The (v) group of mice was each injected with 1 mg/kg of S2P peptide at 1 h before S2P-CeO_2-ASOs administration. The (vi) group of mice was each injected with 1 mg/kg of catalase together with S2P-CeO_2-ASOs administration. 24 h after the injection, distribution of the ASOs in mice was monitored by (1) *ex vivo* fluorescence imaging and (2) histology study. (1) aortas and major organs including heart, liver, spleen, lung and kidney from ApoE$^{-/-}$ mice were collected and visualized with Caliper IVIS Lumina III. The fluorescence signals in each tissue were normalized to the corresponding pre-injection baseline values. (2) 5-μm frozen sections of aortic roots from ApoE$^{-/-}$ mice were fixed in ice-cold acetone for 5 min and then blocked with normal serum. Sections were labeled with unconjugated primary antibodies against smooth muscle α-actin (α-SMA) overnight, followed by Rhodamine (FITC)-conjugated secondary antibody for 1 h. The stained sections were mounted with DAPI-containing mounting medium and then viewed using a TCS SP8 confocal laser scanning microscopy (Leica Co., Ltd. Germany).

***In vivo* silencing efficacy.** ApoE$^{-/-}$ mice (6-8 weeks) fed with the high-fat diet were randomly divided into four groups (n = 3) and given an intravenous injection of (i) PBS, (ii) free ASOs, (iii) CeO_2-ASOs, (iv) S2P-CeO_2-ASOs at 0.5 mg/kg ASOs dose. After three days post-injection, aortas of each group were harvested. To evaluate in vivo silencing efficacy, aortas were homogenized on ice in lysis buffer, respectively. Changes in mTOR expression and LC3II/LC3I ratio were analyzed by western blot following the

same procedure as described above (primary antibody against mTOR: 1:1,000 and LC3B: 1:1,000).

***In vivo* anti-atherosclerotic efficacy.** $ApoE^{-/-}$ mice (6-8 weeks) fed with the high-fat diet were randomly divided into five groups (n = 6) and given an intravenous injection of (i) PBS, (ii) free ASOs, (iii) CeO_2-ASOs, (iv) S2P-CeO_2-ASOs at 0.5 mg/kg ASOs dose. The (v) group of mice was each injected with 4 mg/kg[6] of rapamycin serving as a positive control. The injection was performed twice weekly and the body weights of mice were recorded once weekly. After 12 weeks, all the mice were euthanized and perfused with PBS. The peripheral fat and connective tissue was removed as much as possible. To evaluate the extent of atherosclerotic lesion, two approaches were used. (1) In situ imaging.[7] Aortic arches were dissected under a stereomicroscope (Olympus SZX10, Tokyo, Japan) and imaged by a stereomicroscope-dedicated camera (TrueChrome HD camera, Tucsen, China). (2) *En face* analysis.[8] Whole aortas were opened longitudinally and stained with Oil red O. Photographs of the stained specimens were digitized for data analysis. The luminal lesion surface area was quantified by using NIS-Elements imaging software (Nikon, Japan). Data were expressed as the percentage of the aorta with positive Oil red O staining. To evaluate in vivo toxicity, major organs including hearts, livers, spleens, lungs and kidneys were collected, formalin fixed, paraffin embedded, and haematoxylin&eosin (H&E) stained for histological analysis. Images were captured using a Leica DMI3000 microscope (Leica Microsystems, Wetzlar, GmbH). The body weight of each mouse were monitored and recorded twice a week.

Statistical analysis. Each experiment was repeated three times in duplicate if not stated otherwise. Data were presented as mean ± S.D. Comparisons between groups were analyzed using Student's *t*-test, $P < 0.05$ was considered statistically significant.

Reference

1 H. X. Mai, L. D. Sun, Y. W. Zhang, R. Si, W. Feng, H. P. Zhang, H. C. Liu and C. H. Yan, *J. Phys. Chem. B.*, 2005, **109**, 24380-24385.

2 S. Jang, K. Ohtani, A. Fukuoh, T. Yoshizaki, M. Fukuda, W. Motomura, K. Mori, J. Fukuzawa, N. Kitamoto, I. Yoshida, Y. Suzuki and N. Wakamiya, *J Biol Chem.*, 2009, **284**, 3956-3965.

3 S. Y. Park, Y. Yun, J. S. Lim, M. J. Kim, S. Y. Kim, J. E. Kim and I. S. Kim, *Nat Commun.*, 2016, **7**, 10871.

4 W. Gao, W. Cao, Y. Sun, X. Wei, K. Xu, H. Zhang and B. Tang, *Biomaterials*, 2015, **69**, 212-221.

5 L. Ma, J. Zhong, Z. Zhao, Z. Luo, S. Ma, J. Sun, H. He, T. Zhu, D. Liu, Z. Zhu and M. Tepel, *Cardiovasc Res*, 2011, **92**, 504-513.

6 C. Castro, J. M. Campistol, D. Sancho, F. Sánchez-Madrid, E. Casals and V. Andrés, *Atherosclerosis,* 2004, **172**, 31-38.

7 A. C. MacKinnon, X. Liu, P. W. Hadoke, M. R. Miller, D. E. Newby and T. Sethi, *Glycobiology*, 2013, **23**, 654-663.

8 R. J. Roth Flach, A. Skoura, A. Matevossian, L. V. Danai, W. Zheng, C. Cortes, S. K. Bhattacharya, M. Aouadi, N. Hagan, J. C. Yawe, P. Vangala, L. G. Menendez, M. P. Cooper, T. P. Fitzgibbons, L. Buckbinder and M. P. Czech, *Nat Commun.*, 2015, **6**, 8995.

9 X. Xie, X. e. Yang, T. Wu, Y. Li, M. Li, Q. Tan, X. Wang and B. Tang, *Anal. Chem.*, 2016, **88**, 8019-8025.

Part B: Supplementary tables and figures

Table S1: Sequences of antisense oligonucleotides with modifications used for this study.

Names of oligos	Sequences with modifications (5'->3')
**ASO1**	TCCACTTTTCACAGCACTGC
**ASO2**	ATCTTGCCCTGAGGTTC
**Cy5-ASO1**	Cy5-TCCACTTTTCACAGCACTGC
**Cy5-ASO2**	Cy5-ATCTTGCCCTGAGGTTC

Table S2: Size, zeta potential, ASOs-loading percentage, and number of ASO molecules loaded per NW in various S2P-CeO_2-ASOs formulations.

ASO loaded S2P-PEGylated CeO_2 NW	Mean Size (nM)	Zeta potential (mV)	Encapsulation efficiency (%)	ASO molecules /NW
**S2P-CeO_2-ASO1**	145.4 × 10.5	-11.6 ± 0.4	66.4	395
**S2P-CeO_2-ASO2**	141.2 × 11.1	-12.2 ± 0.2	72.2	482
**S2P-CeO_2-ASOs$_{(1+2)}$**	146.4 × 10.8	-12.0 ± 0.5	70.6	435
**S2P-PEGylated CeO_2 NW**	134.2 × 9.4	4.3 ± 0.3	—	—

Table S3: List of primers used for qRT-PCR

Gene	Primers (5'->3')
**mTOR**	Forward: CCATCCAATCTGATGCTGGA<br>Reverse: GGTGTGGCATGTGGTTCTGT
**GAPDH**	Forward: GGGAAACTGTGGCGTGAT<br>Reverse: GAGTGGGTGTCGCTGTTGA

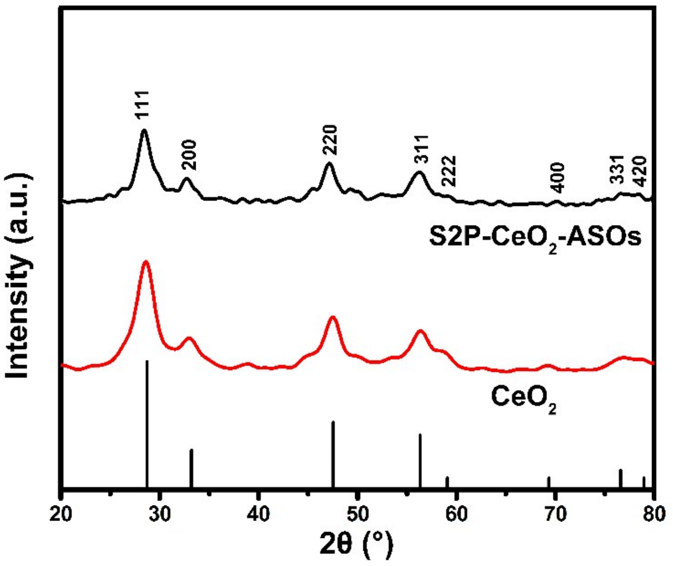

Fig. S1 XRD patterns of CeO_2 NWs and S2P-CeO_2-ASOs nanoplatform.

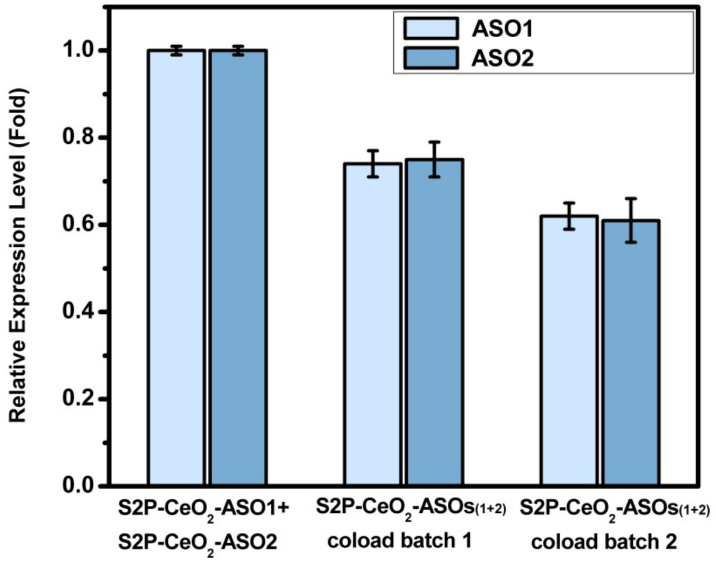

Fig. S2 Quantification of ASO1 and ASO2 loading efficiency in coloaded S2P-CeO_2 nanoplatform by qRT-PCR analysis. No statistical significance was detected in each batch.

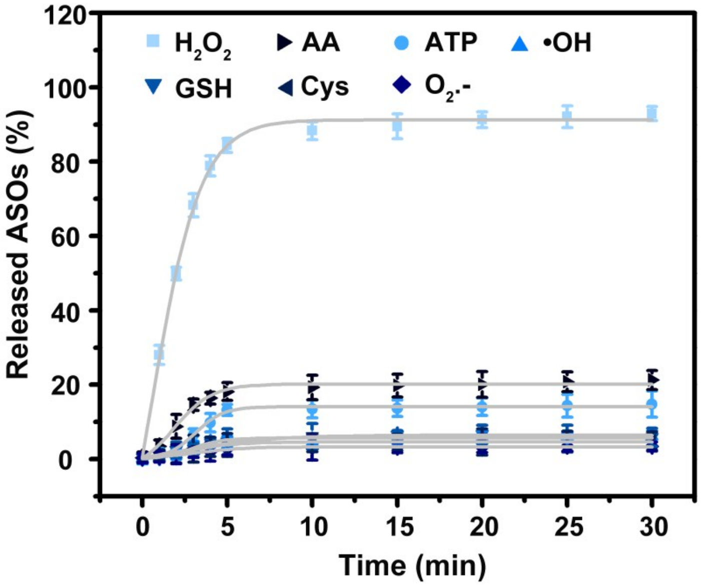

Fig. S3 ASOs released from S2P-CeO_2-ASOs different time points after incubation with H_2O_2, AA, ATP, •OH, GSH, Cys, and $O_2^{\cdot -}$ (100 μM for each).

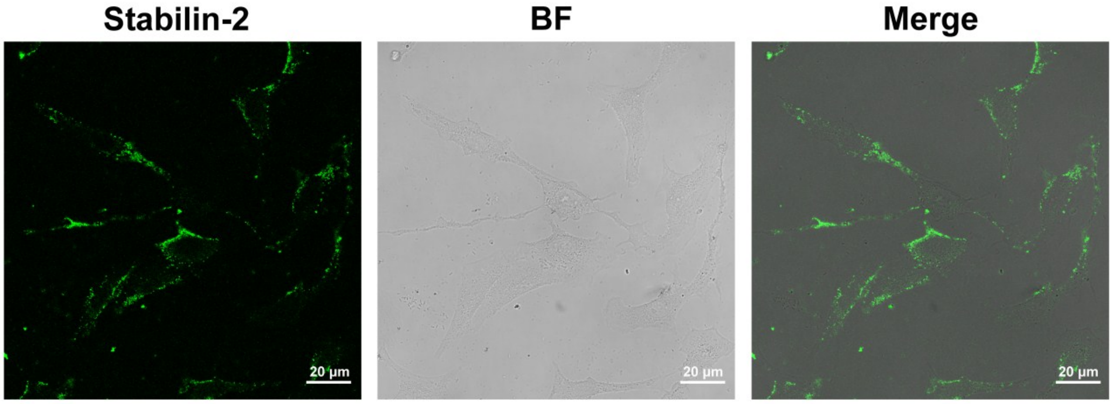

Fig. S4 Immunofluorescence analysis of stabilin-2 expression on VSMCs. From left, stabilin-2 (green, with antirabbit stabilin-2 primary antibody), bright field and merge. Scale bar = 20 μm.

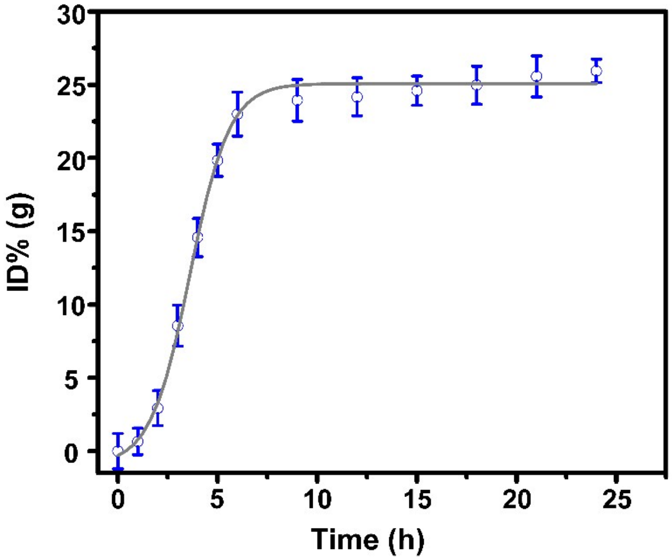

Fig. S5 Uptake of S2P-CeO_2-ASOs recorded by ICP-AES for up to 24 h in VSMCs.

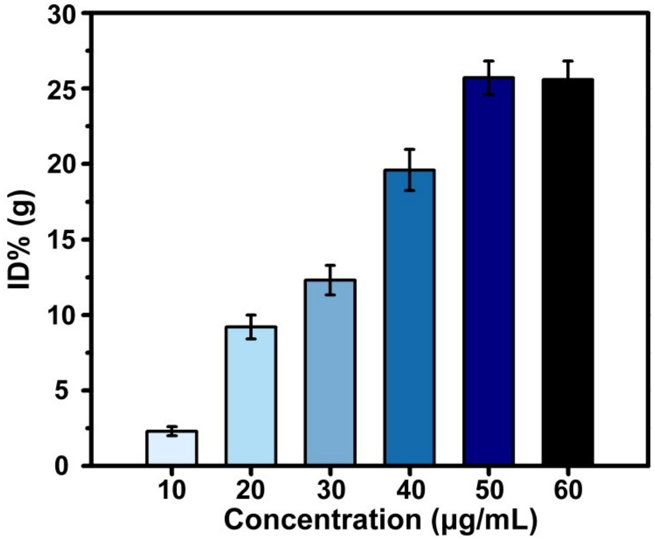

Fig. S6 ICP-AES analysis of VSMCs 6 h after exposure to different concentration of S2P-CeO_2-ASOs.

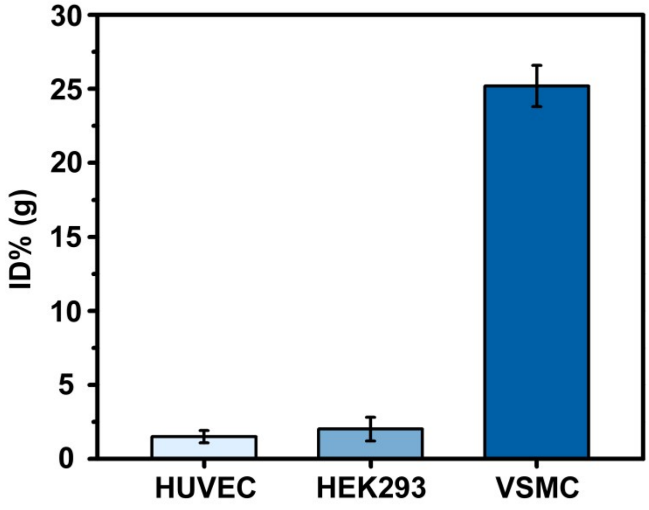

Fig. S7 ICP-AES analysis of HUVEC, HEK293 and VSMC incubated with S2P-CeO_2-ASOs at 37 °C for 6 h at a 50 μg/mL dose.

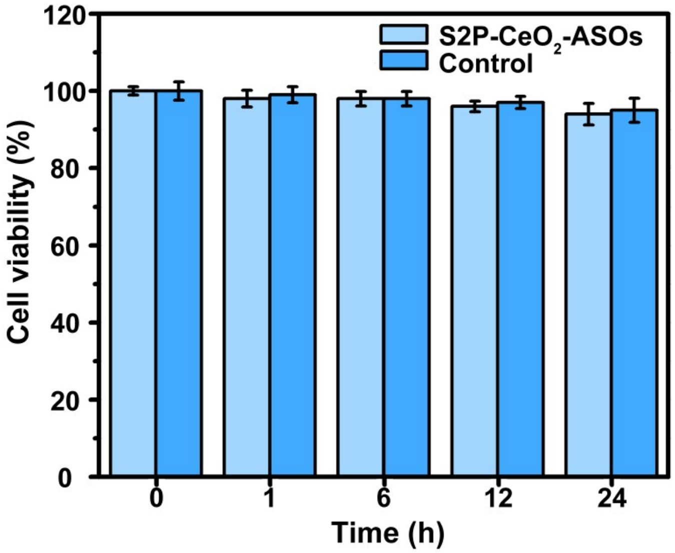

Fig. S8 Viability of VSMCs after S2P-CeO_2-ASOs (50 μg/mL) mediated transfection of ASOs.

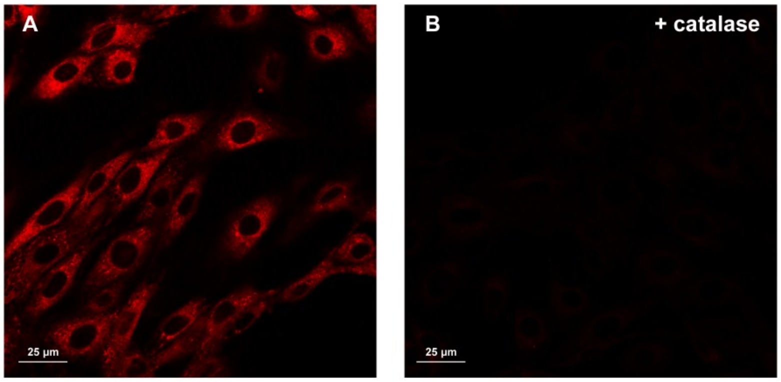

Fig. S9 Confocal fluorescence imaging of endogenous H_2O_2 in VSMCs. (a) Cells stained with a H_2O_2 probe (Mito-NIRHP,[9] 10 μM, 10 min). (b) Cells pretreated with catalase (100 μM, 1 h) and then stained with Mito-NIRHP (10 μM, 10 min). Scale bar = 25 μm.

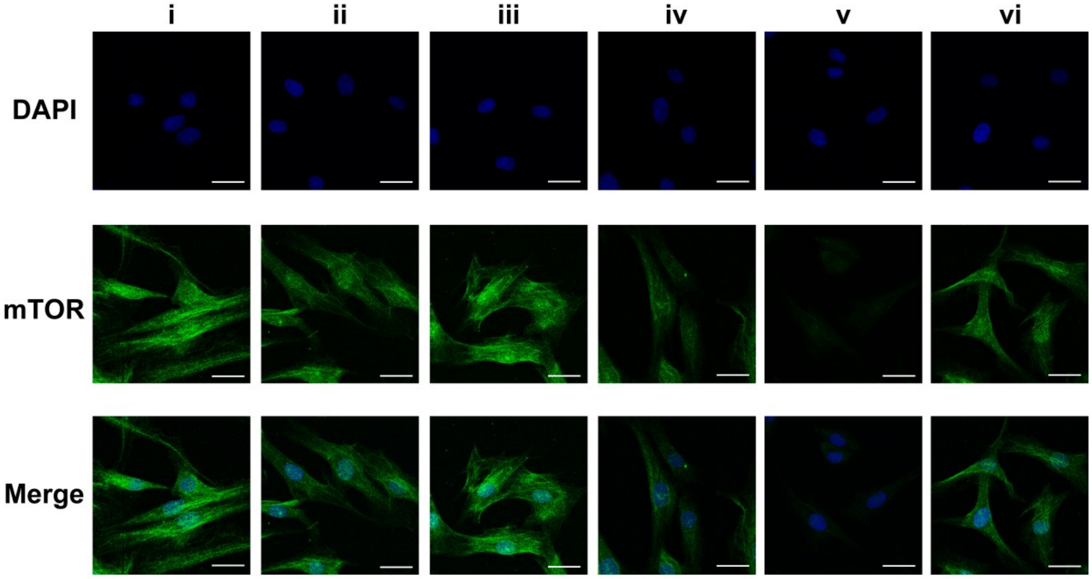

Fig. S10 Immunofluorescence analysis of mTOR expression in VSMCs after treated with (i) blank, (ii) naked ASOs, (iii) S2P-CeO_2, (iv) CeO_2-ASOs, (v) S2P-CeO_2-ASOs and (vi) S2P-CeO_2-ASOs and catalase for 24 h (ASOs dose: 80 ng). From top, DAPI (blue), mTOR (green, with anti-rabbit mTOR primary antibody) and merge. Scale bar = 25 μm.

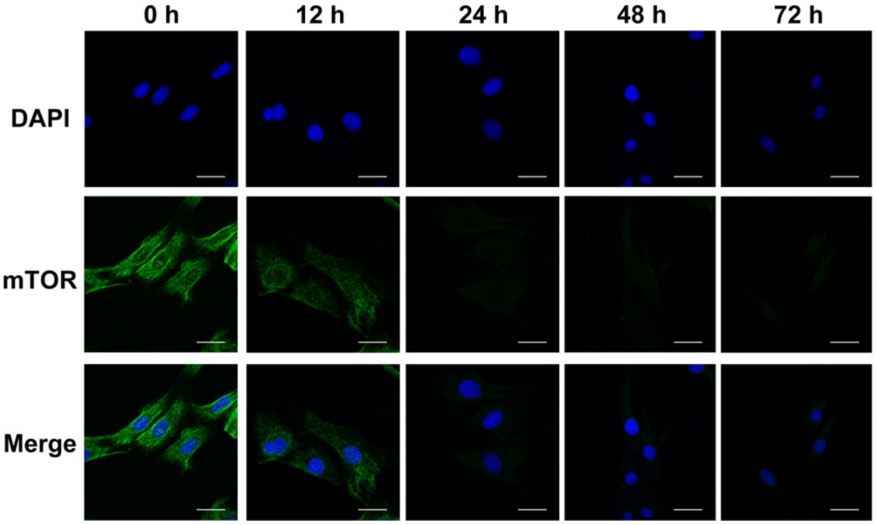

Fig. S11. Immunofluorescence analysis of mTOR expression in VSMCs after treated with S2P-CeO_2-ASOs for 0, 12, 24, 48 and 72 h (ASOs dose: 80 ng). From top, DAPI (blue), mTOR (green, with anti-rabbit mTOR primary antibody) and merge. Scale bar = 25 μm.

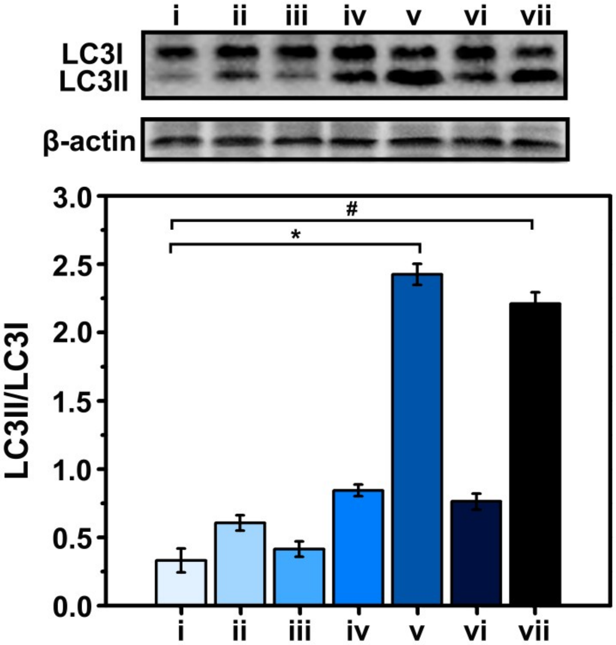

Fig. S12 Western blot analysis of LC3II/LC3I ratio in VSMCs treated with (i) blank, (ii) free ASOs, (iii) S2P-CeO_2, (iv) CeO_2-ASOs, (v) S2P-CeO_2-ASOs, (vi) S2P-CeO_2-ASOs + catalase and (vii) rapamycin for 24 h (ASOs dose: 80 ng; rapamycin: 10 nM). β-actin was used as a loading control. Data are shown as mean ± S.D. (n = 3). $^{*}P < 0.05$ for (v) vs.

(i), $^{\#}P < 0.05$ for (vii) vs. (i).

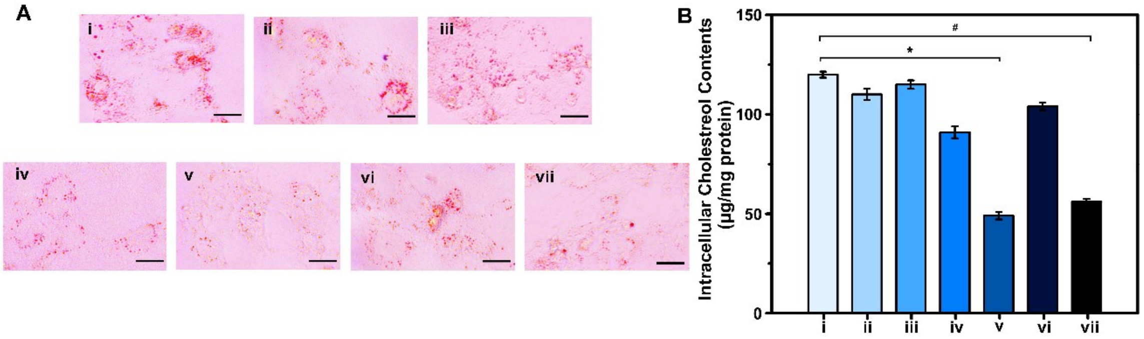

Fig. S13 Downregulation of mTOR expression by S2P-CeO_2-ASOs reduced lipids in VSMCs. VSMCs were cultured in the presence of 60 μg/mL oxLDL for 24 h and treated with (i) blank, (ii) free ASOs, (iii) S2P-CeO_2, (iv) CeO_2-ASOs, (v) S2P-CeO_2-ASOs, (vi) S2P-CeO_2-ASOs + catalase and (vii) rapamycin for 24 h (ASOs dose: 80 ng; rapamycin: 10 nM). (A) Representative pictures of Oil red O staining of intracellular lipid droplets. Scale bar = 25 μm. (B) Quantitative analysis of total cholesterol levels. $^{*}P < 0.05$ for (v) vs. (i), $^{\#}P < 0.05$ for (vii) vs. (i).

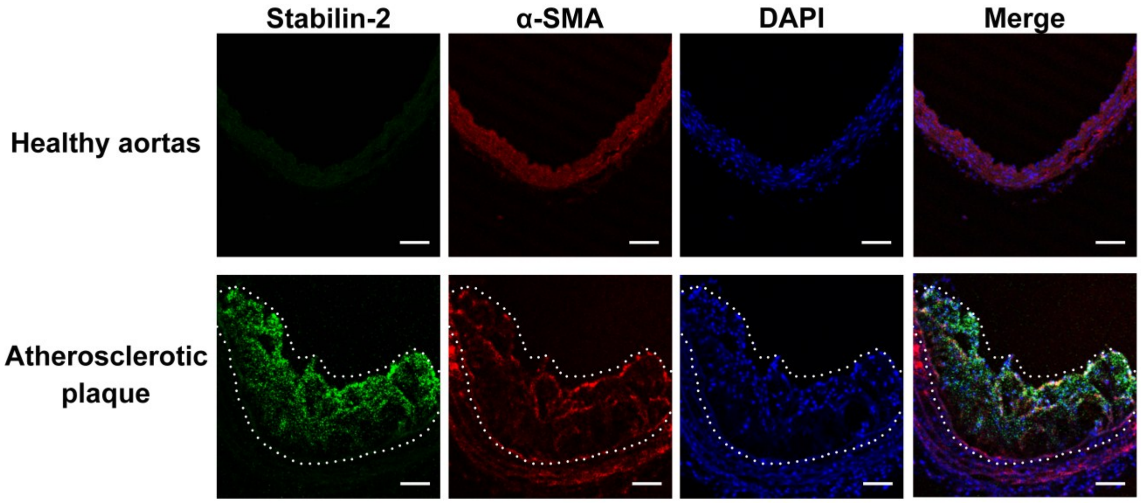

Fig. S14 Immunohistochemical staining of stabilin-2 in aortic arch showing overexpression of stabilin-2 within atherosclerotic plaques. From left, stabilin-2 (green, with antirabbit stabilin-2 primary antibody), α-SMA (red, with antimouse α-SMA primary antibody), DAPI (blue) and merge. The dashed line indicated the plaque border.

Scale bar = 100 μm.

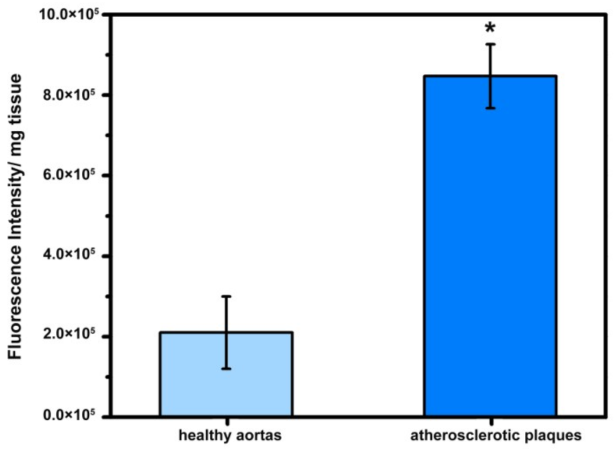

Fig. S15 Increased H_2O_2 production in atherosclerotic plaques. Tissue extracts were isolated from healthy aortas or atherosclerotic plaques, measured by Mito-NIRHP (10 μM, 10 min) and expressed as fluorescence intensity per mg of tissue weight. Data are shown as mean ± S.D. (n = 3), $^{*}P < 0.05$.

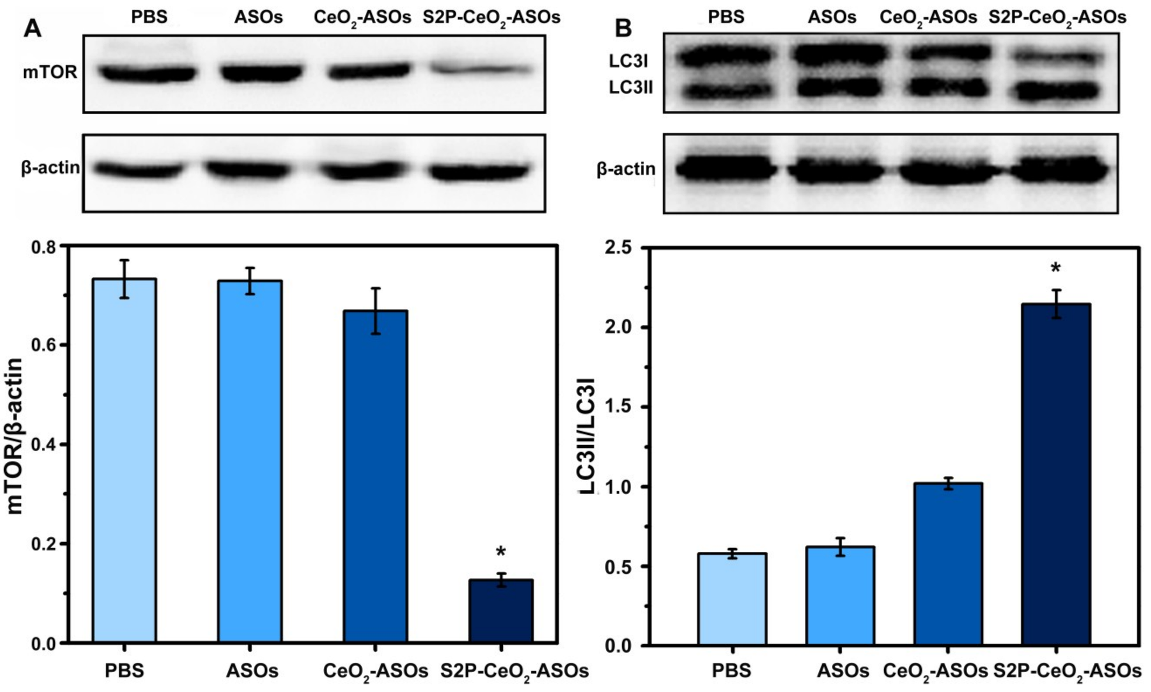

Fig. S16 Western blot analysis of mTOR and LC3II/ LC3I in the aortic lesions from high-fat diet-fed $ApoE^{-/-}$ mice received as indicated treatment. Data are shown as mean ±

S.D. (n = 3). $^{*}P < 0.05$ for S2P-CeO_2-ASOs vs. control.

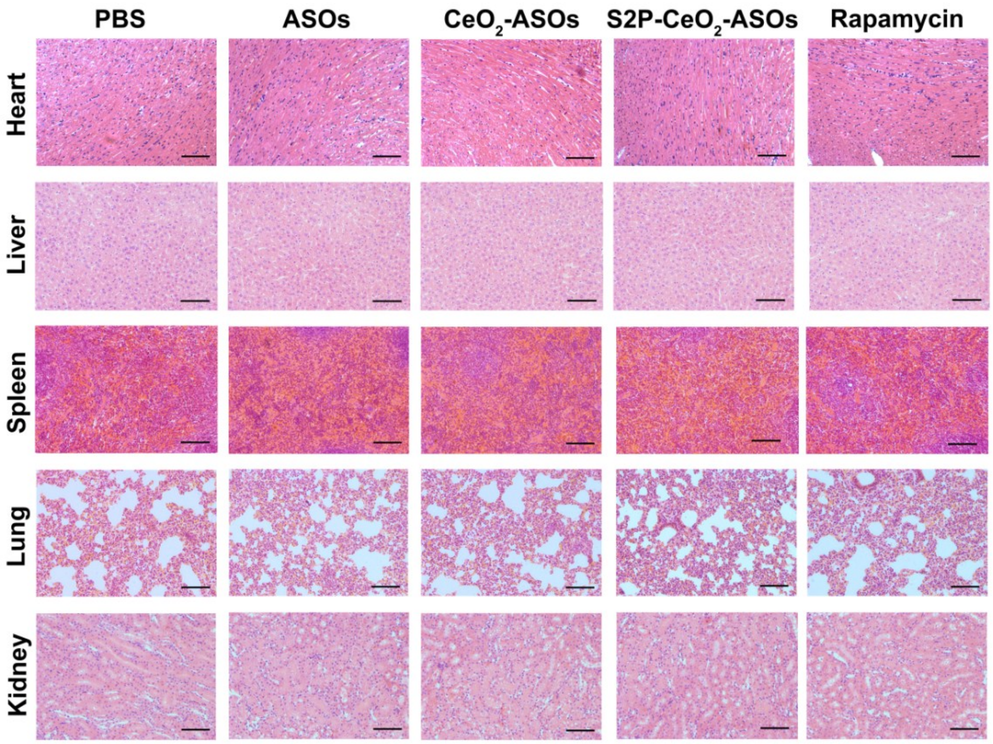

Fig. S17 Representative histology (H&E) images of major organs collected from high-fat diet-fed ApoE$^{-/-}$ mice receiving treatments as indicated after 12 weeks. No noticeable abnormality was found in the heart, liver, spleen, lung, or kidney. Scale bar = 100 μm.

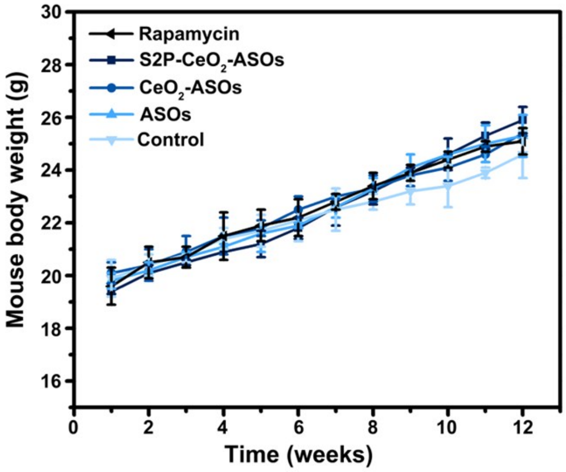

Fig. S18 12-week growth chart of high-fat diet-fed ApoE$^{-/-}$ mice received as indicated treatment. Data are shown as mean ± S.D. (n = 6). No statistical significance was

detected.
